# A novel RFE-GRU model for diabetes classification using PIMA Indian dataset

**DOI:** 10.1038/s41598-024-82420-9

**Published:** 2025-01-06

**Authors:** Mahmoud Y. Shams, Zahraa Tarek, Ahmed M. Elshewey

**Affiliations:** 1 Faculty of Artificial Intelligence, Kafrelsheikh University, Kafrelsheikh, 33516 Egypt; 2 Faculty of Computers and Information, Computer Science Department, Mansoura University, Mansoura, 35561 Egypt; 3 Department of Computer Science, Faculty of Computers and Information, Suez University, P. O. Box 43221 Suez, Egypt

**Keywords:** Diabetes classification, Machine learning, Recursive feature elimination (RFE), Gated recurrent unit (GRU), KNN, Health care, Medical research, Mathematics and computing

## Abstract

Diabetes is a long-term condition characterized by elevated blood sugar levels. It can lead to a variety of complex disorders such as stroke, renal failure, and heart attack. Diabetes requires the most machine learning help to diagnose diabetes illness at an early stage, as it cannot be treated and adds significant complications to our health-care system. The diabetes PIMA Indian dataset (PIDD) was used for classification in several studies, it includes 768 instances and 9 features; eight of the features are the predictors, and one feature is the target. Firstly, we performed the preprocessing stage that includes mean imputation and data normalization. Afterwards, we trained the extracted features using various types of Machine Learning (ML); Random Forest (RF), Logistic Regression (LR), K-Nearest neighbor (KNN), Naïve Bayes (NB), Histogram Gradient Boost (HGB), and Gated Recurrent Unit (GRU) models. To achieve the classification for the PIDD, a new model called Recursive Feature Elimination-GRU (RFE-GRU) is proposed in this paper. RFE is vital for selecting features in the training dataset that are most important in predicting the target variable. While the GRU handles the challenge of vanishing and inflating gradient of the features results from RFE. Several predictive evaluation metrics, including precision, recall, F1-score, accuracy, and Area Under the Curve (AUC) achieved 90.50%, 90.70%, 90.50%, 90.70%, 0.9278, respectively, to verify and validate the execution of the RFE-GRU model. The comparative results showed that the RFE-GRU model is better than other classification models.

## Introduction

Protecting the public from potential health risks and illnesses is a top priority for public health officials. Governments are allocating a significant portion of their GDP to social programs, and some of them, like vaccinations, have directly increased the average lifespan of citizens^1^. Yet, in recent decades, several chronic and hereditary disorders have emerged as major threats to public health policy. Heart diseases, kidneys, and nerves are all made worse by diabetes mellitus, making it a leading cause of death^2^. Diabetics may affect almost every organ in the body^3^. Blurred vision, weariness, hunger, frequent urination, excessive thirst, and changes in body weight are all symptoms of diabetes. Other risk factors for developing diabetes include high or low blood pressure, smoking, and obesity. Millions of individuals all around the globe are afflicted by this illness. As the number of people diagnosed with diabetes continues to rise fast, it has become a worldwide health crisis. Maintaining good health requires prompt attention to any signs of diabetes^4^. By developing insulin resistance, pre-diabetes increases the likelihood that diabetes may be reversed with healthy eating and regular exercise. Major research has shown that long-term diabetes may cause problems. Diseases like macro vascular, cardiovascular, peripheral vascular, stroke, ischemic heart, and chronic heart failure are all major consequences^5^.

Estimating a person’s vulnerability to developing a chronic disease like diabetes is crucial. Early detection of chronic disease may save medical expenses and improve health outcomes. It is important for physicians to be able to make sound judgments about patient care in high-stakes circumstances even when dealing with a patient who is asleep or unable to communicate their condition^6^. Many different predictive, quantitative, and statistical models are used for illness forecasting and diagnosis. Machine learning (ML) is a kind of artificial intelligence (AI) that allows computers to learn automatically by observing their environment and adjusting their behavior accordingly^7^. The most recent advancement in ML has improved computers’ ability to analyze data and make better decisions based on what they find. This includes things like picture recognition and labeling, illness prediction, and more. The goal of machine learning (ML) applications is to teach a computer to achieve human-level performance. The model is trained using a supervised learning method, and its performance is then assessed using testing data^8^. The ML-based algorithms do double duty as classifiers and feature selection methods. In addition to aiding in correct diabetes diagnosis, this solves one of the most pressing issues in the field: how to effectively classify patients to properly assess their risk for developing diabetes. Quadratic discriminant analysis (QDA), linear discriminant analysis (LDA), support vector machine (SVM), naive Bayes (NB), logistic regression (LR), artificial neural network (ANN), Gaussian process classification (GPC), decision tree (DT), Adaboost (AB), random forest (RF), J48, and k-nearest neighbor (KNN) were among the ML-based systems used to categorize and predict diabetes^9^. There is a wealth of data available in today’s healthcare systems. When used intelligently, these statistics have predictive potential. There is a large body of research on diabetes that relies on data collected from a long-term study of Pima Indians in Arizona. Artificial intelligence (AI) technologies and approaches have been developed for early diabetes diagnosis by mining this massive data collection^10^.

In the proposed RFE-GRU model, Recursive Feature Elimination (RFE) and the Gated Recurrent Unit (GRU) architecture interact to create a streamlined, high-performance diabetes classification model that leverages the strengths of both feature selection and sequential data learning while addressing issues commonly associated with gradient vanishing in deep learning. The RFE component serves as a preliminary filter to determine the most relevant features for diabetes classification. By iteratively evaluating feature importance, RFE progressively removes the least significant features, refining the dataset down to the most impactful variables: Glucose, BloodPressure, Insulin, and BMI. This selection not only enhances model interpretability but also reduces input dimensionality, which directly benefits the GRU by simplifying the learning process and improving computational efficiency. With this reduced feature set, the GRU can allocate more computational resources to learning temporal relationships and dependencies within the dataset, instead of processing irrelevant or noisy data. The GRU layer then uses this refined feature set to capture any potential sequential or temporal patterns that may emerge from diabetes-related physiological signals. The GRU’s gated structure is well-suited to model these time dependencies, as it manages information flow through update and reset gates, allowing the network to selectively retain or discard information over time. A well-known issue in traditional Recurrent Neural Networks (RNNs) is the vanishing gradient problem, where gradients shrink as they are back-propagated through layers, leading to difficulties in learning long-term dependencies. GRU addresses this through its gated mechanism, which helps maintain a more stable flow of gradients during training. Specifically, the update gate in the GRU learns to control the degree to which previous information is retained, while the reset gate modulates the incorporation of new information. This design is particularly effective in preventing gradients from vanishing, ensuring that important signals are carried across long sequences without decay.

By combining RFE and GRU, the model minimizes unnecessary data processing, which can contribute to gradient decay when irrelevant or high-dimensional data is included. RFE’s feature selection complements GRU’s architecture by reducing the noise and complexity of input data, effectively enhancing the stability of gradient flow within the GRU model. This hybrid approach results in a robust and efficient model that not only captures critical features for diabetes classification but also mitigates issues related to gradient stability, leading to improved training convergence and predictive accuracy.

The RFE-GRU model addresses several critical research gaps in diabetes classification, particularly in improving feature selection, handling class imbalance, and offering superior computational efficiency compared to existing models. Many previous models fail to properly address irrelevant or redundant features, leading to overfitting and reduced generalization. The RFE method improves this by systematically selecting only the most relevant features, thus reducing dimensionality and enhancing model performance. Furthermore, traditional models often struggle with the class imbalance in the PIDD dataset, but the RFE-GRU model implicitly addresses this issue by emphasizing discriminative features, improving precision and recall for the minority class. Additionally, while many models fail to capture temporal dependencies, GRU (a type of RNN) overcomes challenges like gradient vanishing and explosion, offering better performance in sequential data classification. This model also offers a more generalizable framework that can be extended to other healthcare datasets, and while not using k-fold cross-validation, such techniques could further validate its robustness. Ultimately, the RFE-GRU model offers a more accurate, efficient, and robust approach to diabetes classification, addressing gaps in previous research and improving predictive performance on small, imbalanced datasets like PIDD. The contributions of this work are given as follows:

- Introducing a variety of machine learning methods for classification of diabetes disease. These methods are Recursive Feature Elimination (RFE)- Gated Recurrent Unit (GRU), Logistic Regression (LR), Random Forest (RF), K-Nearest Neighbor (KNN), Naïve Bayes (NB), and Histogram Gradient Boosting (HGB).
- Building a sequential hybrid model based on RFE-GRU to select the most relevant features in the training dataset to facilitate and enhance the prediction results of the target variable.
- Handling the problem of vanishing and inflating gradient of the applied RFE features using GRU.

The rest of this paper is organized as follow. In Sect. 2, the diabetes prediction’s related work is presented. In Sect. 3, the methodology of this research including the preprocessing and ML regressor models is provided. In Sect. 4, the proposed REF-GRU model is presented. Experimental results are discussed in Sect. 5. Finally, the paper conclusion is provided in Sect. 6.

## Related work

Diabetes diagnosis and classification is a difficult undertaking that calls for the knowledge of medical experts. Due to the use of machine learning, deep learning, and ensemble learning techniques, the healthcare sector has recently experienced notable breakthroughs. Numerous methods for categorizing diabetes have been developed, and many of these make use of the PIDD dataset, which has data on 768 cases of female patients across 9 characteristics. We will examine a number of pertinent research on diabetes categorization in this section. A machine learning-based method for diabetes classification, early diagnosis, and prediction was created by Butt et al.^2^. Logistic regression (LR), random forest (RF), and multilayer perceptron (MLP) were three different classifiers that were investigated. They also used the PIDD dataset for predictive analysis utilizing long short-term memory (LSTM), linear regression (LR), and moving averages (MA). MLP had the greatest prediction accuracy (86.08%), while LSTM improved it to 87.26%.

Kaur and Kumari^4^ employed supervised machine learning methods, including radial basis function kernel SVM, linear kernel SVM, k-nearest neighbor (k-NN), multifactor dimensionality reduction (MDR), and artificial neural network (ANN) on the PIDD dataset. Among these methods, the SVM-linear model demonstrated the highest accuracy of 89% for diabetes prediction.

Krishnamoorthi et al.^8^ proposed a novel framework for diabetes prediction using machine learning (ML) techniques. They evaluated popular methods such as decision tree-based random forest (RF) and support vector machine (SVM) learning models. The results of this study have been widely utilized by researchers and practitioners in the medical and scientific communities. The suggested model achieved an accuracy of 83% with a very low error rate. Hrimov et al.^9^ proposed a logistic regression (LR) technique to predict the likelihood of diabetes, achieving an accuracy of 77.06% using a Python program.

Yahyaoui et al.^11^ proposed a diabetes prediction model based on machine learning (ML) and deep learning (DL) approaches. They utilized random forest (RF), support vector machine (SVM), and fully convolutional neural network (CNN) to predict and detect diabetes patients. The experimental findings showed that RF outperformed deep learning and SVM approaches, achieving an overall accuracy of 83.67%.

Sharma et al.^12^ focused on diabetes prognosis using supervised learning methods such as naive Bayes, logistic regression, decision tree, and artificial neural network. Among these methods, logistic regression achieved the best accuracy of 80.43% in determining whether a patient is diabetic or not.

Khanam and Foo^13^ utilized ML algorithms, data mining, and neural network (NN) techniques for diabetes prognosis. They tested seven ML systems on the dataset and found that the model combining logistic regression (LR) and SVM achieved the most effective results. Additionally, they experimented with different epochs and hidden layers for the NN model, concluding that the NN with two hidden layers achieved the highest accuracy of 88.6%.

Hassan et al.^14^ emphasized the importance of early diagnosis of diabetes. They gathered data from the Khulna Diabetes Center, which included 289 cases and 13 variables. The accuracy results of the employed models were 88% for the proposed method, 86.36% for XGBoost, and 86.36% for Random Forest. Howlader et al.^15^ utilized ML algorithms to classify individuals with Type 2 diabetes. They employed various feature selection methods and classification techniques, finding that the Generalized Boosted Regression model achieved the highest accuracy of 90.91%.

Çalisir and Dogantekin^16^ introduced a linear discriminant analysis (LDA) with Morlet wavelet support vector machine (MWSVM) classifier for automatic diabetes diagnosis. They achieved an accuracy of 89.74% using the PIDD dataset. Dadgar and Kaardaan^17^ presented a hybrid technique combining the UTA algorithm and a two-layer neural network for diabetes prediction. The method utilized feature selection using the UTA algorithm and neural-genetic prediction, achieving a predictive accuracy of 87.46% on the PIDD diabetes dataset.

Chen et al.^18^ developed a prediction model to assist in the prognosis of Type 2 diabetes. They employed K-means for dimensionality reduction of the PIDD dataset and J48 decision tree as a classifier, achieving an accuracy of 90.04%. Haritha et al.^19^ proposed a hybrid model based on Firefly and Cuckoo Search algorithms. They utilized the PIMA dataset with a K-nearest neighbor (KNN) classifier and Cuckoo fuzzy KNN algorithm, achieving an accuracy of 81.00%.

In order to predict diabetes, Zhang et al.^20^ proposed a multi-layer feed-forward neural network that used risk indicators from the PIDD dataset. The Levenberg-Marquardt training method was used to train the network, which resulted in an accuracy rating of more than 80%. On the PIDD dataset, Benbelkacem and Atmani^21^ used random forest (RF) with 100 trees to classify diabetes with an accuracy ranging from 70.00 to 80.00%. Khanwalkar and Soni^22^ utilized the PIDD dataset with a Sequential Minimum Optimization (SMO) algorithm based on quadratic programming, achieving an average accuracy of 77.35% for predicting diabetes.

Patra and Kuntia^23^ introduced a standard deviation KNN (SDKNN) algorithm for diabetes classification on the PIDD dataset. The algorithm utilized the standard deviation of KNN features to calculate the distance between the train and test data, achieving an accuracy of 83.76% for the modified weighted SDKNN. Bhoi et al.^24^ employed various supervised learning techniques, including classification tree (CT), random forest (RF), neural network (NN), SVM, KNN, naive Bayes (NB), AdaBoost (AB), and logistic regression (LR) on the clinical PIDD dataset to predict diabetes in Pima Indian women. They evaluated the accuracy, precision, recall, F1 score, and AUC of these models.

Ramesh et al.^25^ proposed an end-to-end healthcare monitoring approach for controlling diabetes and detecting risk cases based on the PIDD dataset. They achieved sensitivity, specificity, and accuracy rates of 87.20%, 79.00%, and 83.20%, respectively. Salem et al.^26^ presented a preprocessing stage for the PIDD dataset, including normalization, elimination of outliers, imputation of missing values, and feature selection. They used a fuzzy-KNN classifier to classify the resulting features, considering the uncertainty values of the membership function. Through a grid search process, they obtained tuning values for the fuzzy-KNN and achieved an accuracy of 90.63%. Studies should concentrate on using standardized datasets, exploring a wide range of machine learning algorithms, providing a detailed description of the methodology used, and comparing the performance of various algorithms using suitable evaluation metrics to increase the quality and reliability of diabetes prediction models. A list of research that have utilized various machine learning methods and datasets to predict diabetes is included in Table 1.

**Table 1:** The comparative study for the most recent approaches and recommendations.

Author	Methodology	Dataset used	Advantages	Recommendations
Butt et al.^2^	MLP, RF, LR, LSTM, LR, MA	PIDD dataset	MLP achieved 86.08% accuracy, LSTM improved to 87.26%	Further investigation of LSTM and other predictive models
Kaur and Kumari^4^	RBF-SVM, Linear SVM, k-NN, MDR, ANN	PIDD dataset	SVM-linear achieved 89% accuracy	Focus on SVM-linear model for diabetes prediction
Krishnamoorthi et al.^8^	Decision tree-based RF, SVM	Not specified	Achieved 83% accuracy with low error rate	Utilize proposed model for diabetes prediction in medical and scientific fields
Hrimov et al.^9^	LR	Python program	Achieved 77.06% accuracy	Explore other classification algorithms for improved accuracy
Yahyaoui et al.^11^	RF, SVM, CNN	Not specified	RF outperformed DL and SVM with 83.67% accuracy	Investigate ensemble methods combining RF and DL for better results
Sharma et al.^12^	Naive Bayes, LR, Decision tree, ANN	Not specified	Logistic regression achieved 80.43% accuracy	Further evaluation of other supervised learning methods
Khanam and Foo^13^	ML algorithms, NN	Not specified	Combination of LR and SVM achieved 88.6% accuracy	Investigate different NN architectures for improved accuracy
Hassan et al.^14^	Proposed method, XGBoost, RF	Khulna Diabetes Center	Achieved 88% accuracy	Focus on proposed method and compare with XGBoost and RF
Howlader et al.^15^	Generalized Boosted Regression	Not specified	Achieved 90.91% accuracy	Explore feature selection methods and compare with other classification models
Çalisir and Dogantekin^16^	LDA, MWSVM	PIDD dataset	Achieved 89.74% accuracy	Investigate the performance of other classifiers with LDA and wavelet support
Dadgar and Kaardaan^17^	UTA algorithm, NN	PIDD dataset	Achieved 87.46% predictive accuracy	Investigate different feature selection algorithms and compare with UTA
Chen et al.^18^	K-means, J48 decision tree	PIDD dataset	Achieved 90.04% accuracy	Consider other dimensionality reduction techniques and compare classifiers
Haritha et al.^19^	Firefly algorithm, Cuckoo Search	PIDD dataset	Achieved 81.00% accuracy	Explore other hybrid models and compare with KNN and fuzzy KNN
Zhang et al.^20^	Multi-layer feed-forward NN	PIDD dataset	Achieved accuracy above 80%	Investigate other training algorithms and compare with NN
Benbelkacem and Atmani^21^	RF	PIDD dataset	Achieved accuracy between 70.00% and 80.00%	Explore other classification algorithms for improved accuracy
Khanwalkar and Soni^22^	SMO algorithm based on quadratic programming	PIDD dataset	Achieved average accuracy of 77.35%	Investigate other optimization algorithms for better performance
Patra and Kuntia^23^	Standard deviation KNN (SDKNN)	PIDD dataset	Achieved 83.76% accuracy for modified weighted SDKNN	Evaluate other distance metrics and compare with KNN
Bhoi et al.^24^	CT, RF, NN, SVM, KNN, NB, AB, LR	Clinical PIDD dataset	Evaluation of multiple supervised learning techniques	Consider precision, recall, F1 score, and AUC when comparing models
Ramesh et al.^25^	End-to-end healthcare monitoring	PIDD dataset	Sensitivity: 87.20%, Specificity: 79.00%, Accuracy: 83.20%	Further investigation of risk detection cases and their impact
Salem et al.^26^	Preprocessing, fuzzy-KNN classifier	PIDD dataset	Achieved 90.63% accuracy	Optimize tuning values for fuzzy-KNN classifier and explore other classifiers

## Methodologies

**Fig. 1:**
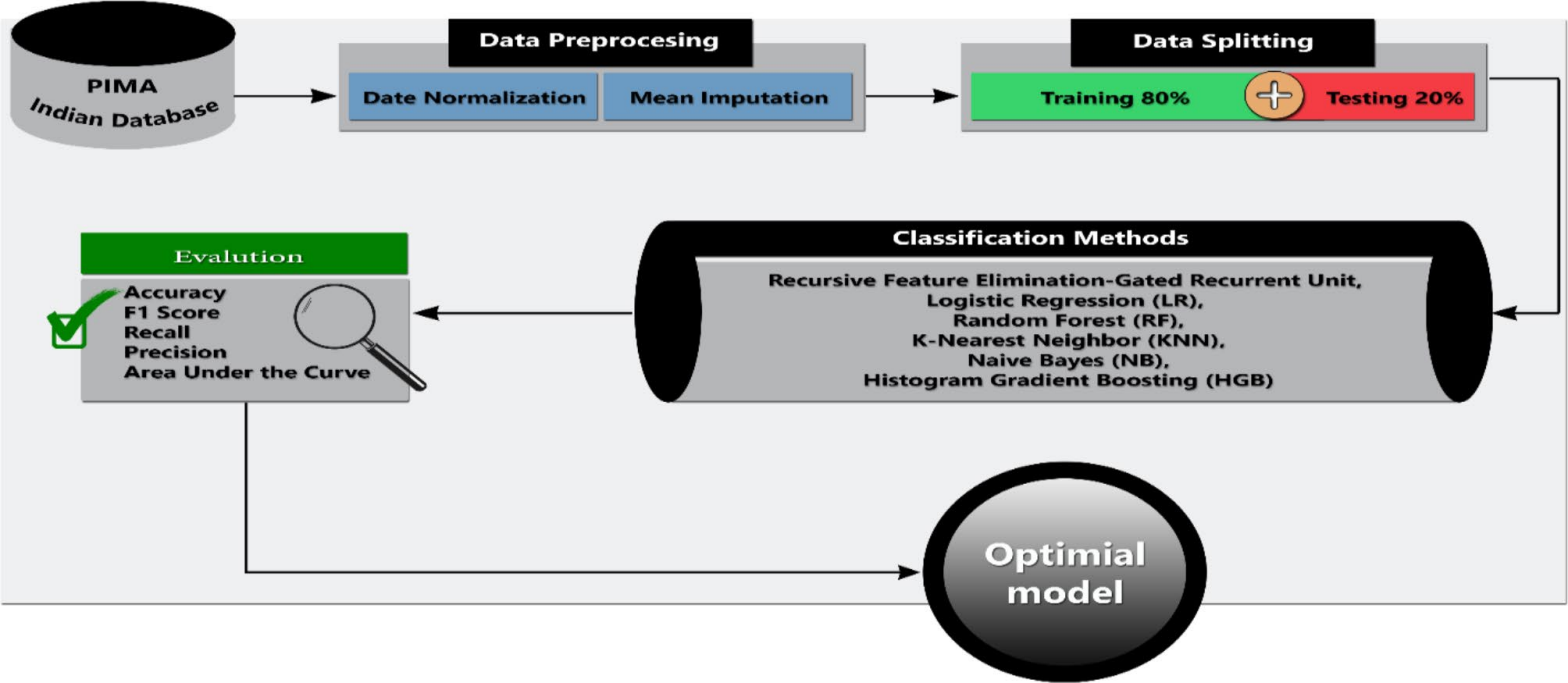
The general block diagram of the proposed methodology.

It is true that ML algorithms are now helpful in illness identification, however older research models were less accurate. Thus, it is necessary to develop new methods that can combine the precision of several algorithms for accurate illness diagnosis. For this purpose, a ML-based system is employed in this paper to classify the diabetes disease. The overview of the proposed ML-based systems has been shown in Fig. 1. The proposed methodology contains numerous processes such as diabetes data preprocessing using data normalization and mean imputation, noise data removal, feature extraction, selection, disease classification, and evaluating results using several predictive evaluation metrics including precision, recall, F1 score, accuracy, and Area Under the Curve (AUC). Optimized techniques are used to predict the diabetes sickness, such as Recursive Feature Elimination (RFE)- Gated Recurrent Unit (GRU), Logistic Regression (LR), Random Forest (RF), K-Nearest Neighbor (KNN), Naïve Bayes (NB), and Histogram Gradient Boosting (HGB). The obtained results illustrated that the RFE-GRU model performed better than other classification models and several studies used PIDD for classification.

The GRU model utilized 64 hidden units, a batch size of 32, a learning rate of 0.01, 200 epochs, and an Adam optimizer to enhance convergence efficiency. The GRU was further optimized with 8 time steps to capture temporal dependencies within the data, and a sigmoid activation function in the output layer to produce probabilistic predictions suitable for classification tasks.

To rigorously evaluate the RFE-GRU model, we compared its performance against Logistic Regression (LR), Random Forest (RF), Histogram-Based Gradient Boosting (HGB), K-Nearest Neighbors (KNN), and Naïve Bayes (NB) classifiers. The LR model was configured with L2 penalty (ridge regularization) and fit_intercept = true to calculate the intercept term, optimizing regularization. For the RF model, we set n_estimators to 100, providing a robust number of decision trees in the ensemble for stable performance. The HGB model utilized a learning rate of 0.01, which controls the step size during gradient correction to avoid overfitting while promoting gradual convergence. In the KNN model, we set n_neighbors to 5 with Euclidean distance as the metric, effectively balancing model simplicity and performance by selecting the nearest neighbors. Lastly, the NB model used alpha = 0.5 (Laplace smoothing) to handle cases with zero probability and fit_prior = true to adjust class priors based on the dataset distribution.

To ensure comprehensive model evaluation, we assessed performance metrics for each model, including accuracy, precision, recall, F1 score, and AUC. These metrics allowed us to thoroughly compare each classifier’s strengths and limitations. The configuration of these hyperparameters, underscoring the importance of precise parameter tuning to maximize each model’s accuracy and predictive power for the task of diabetes classification.

### Data preprocessing

Missing data imputation and outlier removal are instances of data preprocessing methods that may be used to enhance the goodness of the raw data. Preprocessing approaches for missing data, outlier identification, data minimization, data conversion, data scaling, and data segmentation are outlined in terms of their applicability^27^. This article utilizes the mean imputation and data normalization operations for preprocessing the PIDD.

#### Mean imputation

**Fig. 2:**
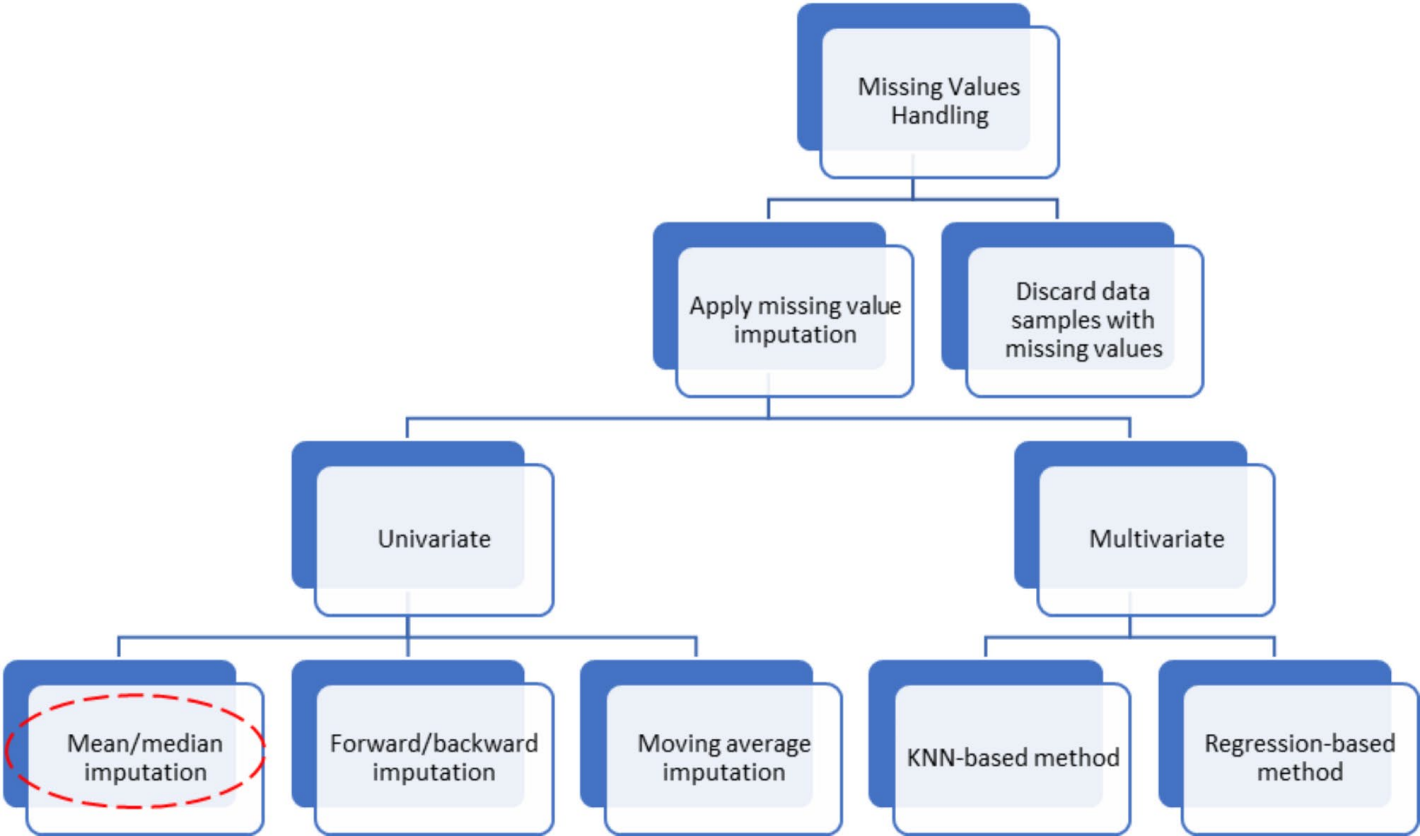
The hierarchal taxonomy of missing value imputation.

The primary strategies for dealing with missing values are investigated in Fig. 2, by which the creating of operational data are performed. Moreover, in this study, we concentrate on mean imputation, which replaces missing values with the variable’s mean^27^.

Mean imputation was chosen for handling missing values in the dataset due to its simplicity and efficiency, particularly given the nature of the PIMA Indian Diabetes Dataset (PIDD). The choice of mean imputation provides a straightforward and computationally inexpensive method for filling in gaps in the data, ensuring consistency without introducing significant variance, which could potentially skew the model’s performance^28–30^.

Alternative imputation methods, such as median or KNN imputation, were considered, but mean imputation was ultimately selected based on its balance between computational efficiency and reliability in preserving the dataset’s overall distribution^31,32^. While median imputation could mitigate the impact of outliers, it may not significantly alter results in datasets with normally distributed features, such as PIDD. KNN imputation, while potentially more accurate, can introduce additional computational complexity and time, which may not justify its benefits in this case^33^. Ultimately, mean imputation was deemed the most practical choice to maintain simplicity while providing reliable data for the classification models^34^.

#### Data normalization

Normalization is an original data procedure that rescales or transforms information such that each characteristic contributes uniformly. It tackles the two most significant data issues that have been a barrier to the progress of machine learning methods, namely the existence of dominating features and outliers. Numerous methods for data normalization within a given range using statistical measures derived from raw data have been explored^35^. These normalization methods are as follows:

- Standard Deviation and Mean (e.g., Pareto Scaling, Mean Centered, Z-score Normalization, Variable Stability Scaling, Power Transformation).
- Minimum–Maximum Value (e.g., Max Normalization, Min–Max Normalization).
- Decimal Scaling Normalization.
- Median and Median Absolute Deviation Normalization.
- Sigmoidal Normalization (e.g., Hyperbolic Tangent, Logistic Sigmoid).
- Tanh Based Normalization.

The input parameters of proposed supervised learning techniques are normalized in this research to stabilize the learning strategy. The results are normalized to a range of (0, 1) using the min-max method.

### Machine learning algorithms

#### Logistic regression (LR)

LR is the most extensively utilized experiential approach in several scientific domains, particularly in environmental research. In LR, the occurrence possibility of a phenomena is assessed between 0 and 1, and the predictor variables’ normality is not assumed. LR is a form of multiple regression with a discrete dependent parameter^36^. In logistic stepwise regression (LSR), LR is among the most frequently used mathematical approaches. It defines the link between a category parameter and several dependent variables that might be binary, continuous, or discrete variables. The logistic function Li is used to calculate the LR as shown in Eq. (1)^37^.

1 $$L = \frac{{\exp \left( v \right)}}{{\left( {1 + \exp \left( v \right)} \right)}}$$

where P denotes the probability associated with a certain observation, and v can be expressed as in Eq. (2):

2 $$v = \beta _{0} + \beta _{1} X_{1} + \beta _{2} X_{2} + \cdots + \beta _{n} X_{n}$$

where $\:{\beta\:}_{0}\:$signifies the algorithm’s intercept, βi indicates the coefficient of independent parameters contribution Xi, and n is the conditioning factors number.

#### K- nearest neighbor (KNN)

Among approaches for machine learning, the KNN is often used to categorize the standard datasets. Even compared to the complicated machine learning algorithms, this straightforward and simple-to-implement technique produces amazing results. The increasing retrieval of data in a variety of media, including text, photographs, music, and video, has prompted recent interest in KNN. However, the success of KNN is highly dependent on several parameters, such as the distance measure and k parameter selection. The square root of the Euclidean distance is calculated by taking the total of the squared differences that exist between an existing point (xi) and a new point (x), The calculation is given by Eq. (3), which is the most often used distance measurement as in Eq. (3)^38^.

3 $$d\left( {x,x_{i} } \right) = \sqrt {\sum\nolimits_{{i = 1}}^{n} {\left( {x_{j} - x_{{ij}} } \right)^{2} } }$$

**Fig. 3:**
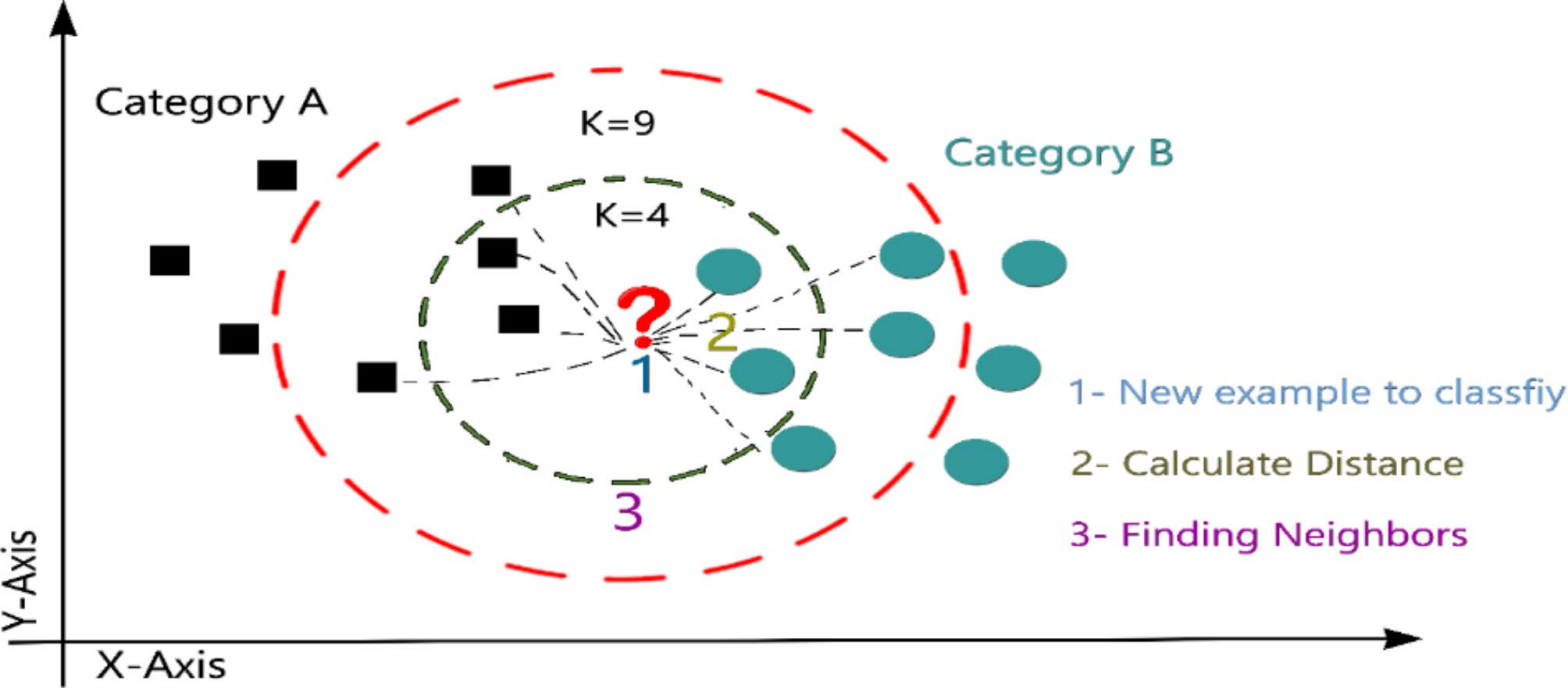
KNN classification example.

Data for training and testing is loaded into the KNN algorithm, which then assigns a category to each test point. It is computed for every point in the dataset and saved in a list ordered by increasing distance while the halting state is not yet reached. After that, the K spots with the shortest distance between them will be chosen. Training samples from a smaller neighborhood will be utilized to make predictions when k is reduced, which reduces approximation error but increases estimation error. Classification models with a low k value are oversimplified. Cross-validation is often employed to find the optimal k value in real-world applications since the k value typically takes on a modest value. K occurrences in these categories should be determined, as well. Grouped by the number of occurrences at point K, the test samples were sorted into the appropriate groups^39^. An example of KNN classification is presented in Fig. 3.

#### Naïve Bayes (NB)

The Nave Bayes technique is based on the Bayes’ theorem and a rudimentary hypothesis about the absence or presence of a certain characteristic. No additional feature class is required for this feature class to exist or not exist. The theoretical features of the NB classifier may be summarized as follows: Random variables for the collection of predictors and the goal characteristic may be represented using the x1-xn formula: (*x*_*1*_, *x*_*2*_,*… x*_*n*_) (with *K* values). One further feature to consider when making predictions is Y, which has K potential values symbolized by (C_1_,.C_K_). X_i_ and Y are frequently referred to as independent and dependent variables, respectively^40^. As a result, Bayes’ theorem can be used as in Eq. (4).

4 $$R(Y = C_{K} |X = x) = \frac{{R\left( {Y = C_{K} } \right)\prod\nolimits_{{i = 1}}^{n} {R(X_{i} = x_{i} |Y = C_{K} )} }}{{R(x_{1} \ldots ,x_{n} )}}$$

These computations are carried out on the log scale to prevent arithmetic underflow (when n > > 0), and the category of the greatest log-posterior possibility is selected to be the result of forecasting, which is identical to anticipate (…, category = “class”), then the result is determined as in Eq. (5)

5 $$C^{ \wedge } = \arg \max _{{k \in \left\{ {1, \ldots ,k} \right\}}} (\log R\left( {Y = C_{K} } \right) + \sum\nolimits_{{i = 1}}^{n} {\log R\left( {X_{i} = x_{i} |Y = C_{K} } \right)}$$

#### Random forest (RF)

**Fig. 4:**
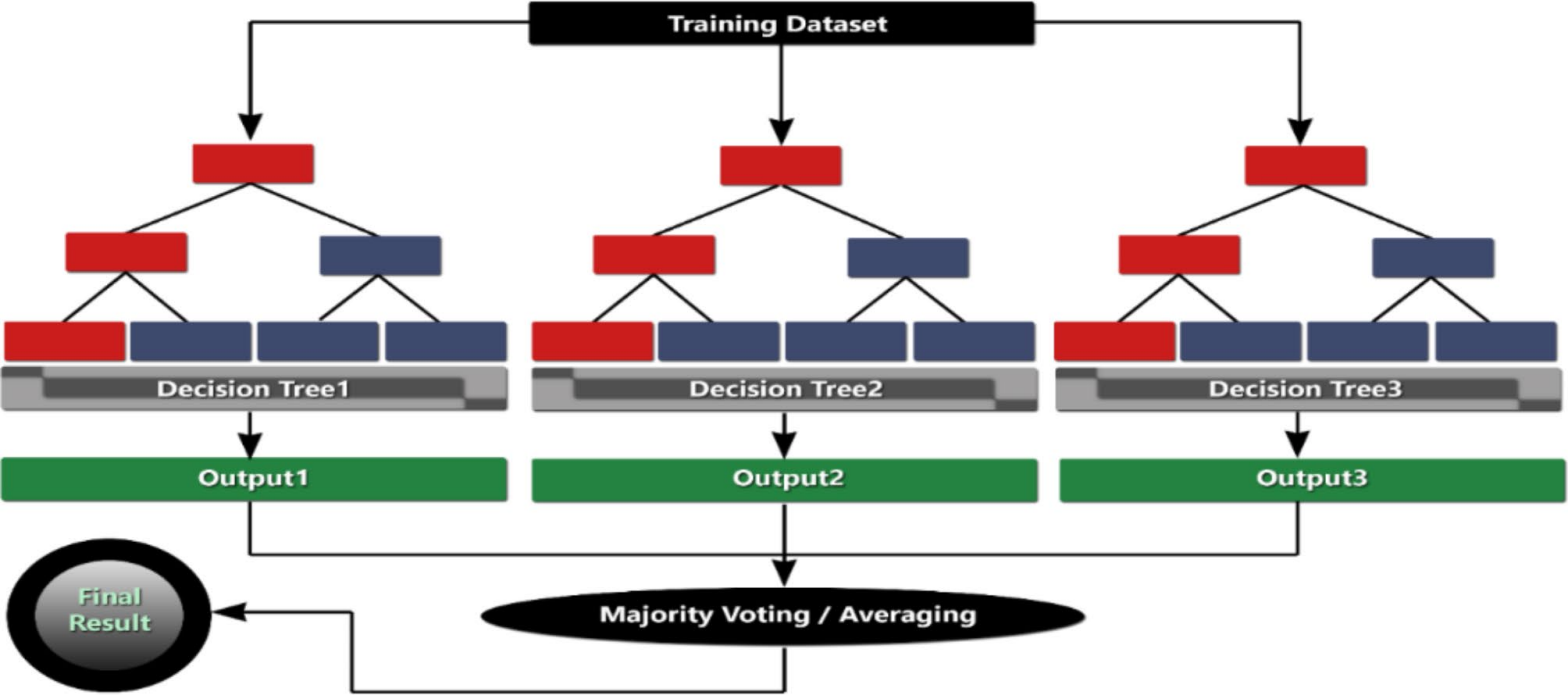
Random Forest structure.

Bierman et al.^41^ proposed an ensemble method, known as random forest (RF), that combines many trees focused on the ensemble learning idea. A decision tree, one of the learning techniques used in integrated learning approaches in machine learning, serves as the foundation of the random forest algorithm. The RF method has good accuracy and is often utilized in biological sequence alteration studies. The two-classification issue is addressed by RF, which may be seen as a unique bagging technique. The original unit decision tree is employed as a model in this procedure. This is the precise procedure: First, a guided technique is used to produce X training sets. Decision trees are then built to match each training set. The optimal answer is identified in a randomly chosen piece of the feature, which is then given to the node and split when the decision tree member identifies the feature that has to be divided. The integration technique is the fundamental idea behind the RF method. In order to prevent overfitting, the training data is transformed into a sampling matrix using the bagging and integration ideas, which are comparable to the sampled features and samples that prevents overfitting by converting the training data into a sampling matrix^42^. The structure of the Random Forest is illustrated in Fig. 4.

#### Histogram gradient boosting (HGB)

The gradient boosting method has been modified by the HGB. The approach is based on building a group of decision trees that reduce the loss function one by one. The alteration is distinct since the input data is discretized first. This enables the technique to leverage integer data representations (histograms) rather than depend on ordered continuous data to form trees, considerably reducing the number of examined division points. This algorithm’s capacity to adjust its parameters as fresh data is provided rather than retraining is a benefit of this algorithm^43^. The fact that this technique offers native support for missing data in the database is one of the most important characteristics that it defines. During training, the tree grower will learn at each split point, depending on the potential gain, the instances with missing values should go to the left child or the right child. In the process of prediction, samples that are lacking values are therefore given to either the right or left child. In the event that the dataset contains no instances of missing values, samples with missing values will be matched to the majority of child samples^44^. HGB is superior to the Gradient Boosting Machine in terms of both the amount of memory it uses and the speed with which it processes data^45^.

## The proposed RFE-GRU method

Following a systematic process, RFE assesses the significance of each feature, iteratively eliminating the least important features, and recalculating feature importance. This iterative approach enables RFE to prioritize the most influential features and gradually build a ranked list. By obtaining this ranked list, RFE provides valuable insights into the relative importance of each feature, enabling data scientists and researchers to make informed decisions during feature selection. In this paper, we present Recursive Feature Elimination (RFE) with Gated Recurrent Unit (GRU) sequential hybrid model to develop and improve the performance by selecting the most relevant feature of the PIDD dataset, along with using intelligent models (LR, RF, KNN, NB, and HGB) for comparison with the proposed methodology. For evaluation of the suggested algorithms’ dependability, the area under the curve (AUC) and several other validation methods such as F1-score, recall, accuracy, and precision are compared.

### Recursive feature elimination (RFE)

**Fig. 5:**
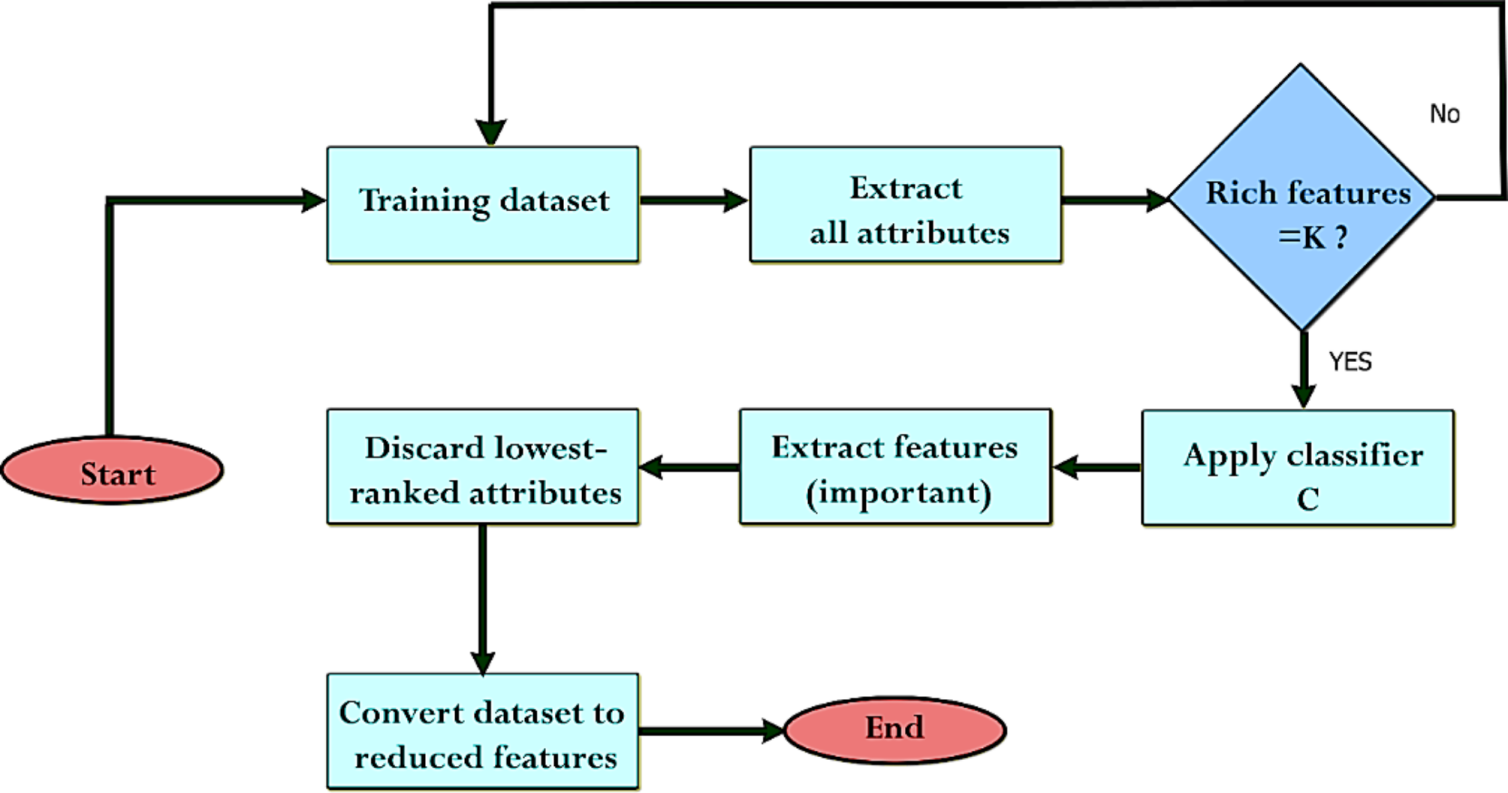
Recursive Feature Elimination steps.

Guyon et al.^46^ introduced Recursive Feature Elimination (RFE), a novel and powerful embedded feature selection approach. RFE is a greedy technique based on approaches of feature ranking. RFE begins with a full set and then removes, one by one, the least significant traits in order to identify the most essential ones^47^. The main technique of RFE uses a different machine learning algorithm. The RFE algorithm uses this selected machine learning algorithm as a wrapper and makes use of it to help in feature selection. Finding a subset of features that are most pertinent to the specified machine learning job is the aim of RFE. Beginning with every feature included in the training dataset, RFE includes iteratively removing less significant features until the required number of features is left. The selected machine learning method is used throughout each iteration to assess the significance of each characteristic, and the least important ones are disregarded. Until the necessary number of features is obtained, this recursive procedure is repeated. The number of features that must be chosen during the feature selection process is controlled by the “Feature Set Size” option in RFE. The final subset of characteristics that the method seeks to keep following repeated elimination stages is chosen by providing the feature set size limit as part of RFE’s setup. RFE starts off with every feature in the dataset and gradually removes the least significant ones until it reaches the desired feature set size. Overfitting is limited by the feature set size selection^48^. RFE improves the model’s prediction ability and enables improved generalization of previously unobserved data by concentrating on the most important characteristics. The chosen features are extremely important for increasing the model’s precision, interpretability, and effectiveness since they capture the key relationships and patterns in the data. Machine learning models that are more effective and efficient and specifically adapted to the particular problem domain may be created using RFE’s ability to prioritize and delete features according to their relevance. Figure 5 is a step-by-step flowchart of the selection criteria for ideal characteristics of RFE selection criteria^49^.

Recursive Feature Elimination (RFE) was chosen over other feature selection techniques due to its ability to iteratively eliminate the least significant features, allowing the model to focus on the most relevant attributes for the specific classification task^50^. Unlike dimensionality reduction methods like Principal Component Analysis (PCA), which transforms features into a new set of uncorrelated variables, RFE maintains the original feature set while identifying the most relevant ones^51^. This approach enhances model interpretability because it preserves the semantic meaning of the features, making it easier to understand which specific attributes contribute to the model’s predictions^52^. RFE also outperforms methods like mutual information in certain cases, as it not only considers the statistical relationships between features and target variables but also evaluates feature importance based on the performance of a specified classifier^53^. By using a chosen machine learning algorithm as a wrapper, RFE ensures that the selected features are optimized for the model’s predictive performance, leading to better classification results and generalization^54^. In this study, RFE improved model performance by reducing the risk of overfitting. By focusing only on the most critical features, RFE enables a model that is less complex, computationally efficient, and more robust on unseen data. This targeted selection of features captures key relationships and patterns in the dataset, ultimately leading to higher accuracy and predictive reliability, as observed in the RFE-GRU model compared to other classifiers.

### Gated recurrent unit (GRU)

**Fig. 6:**
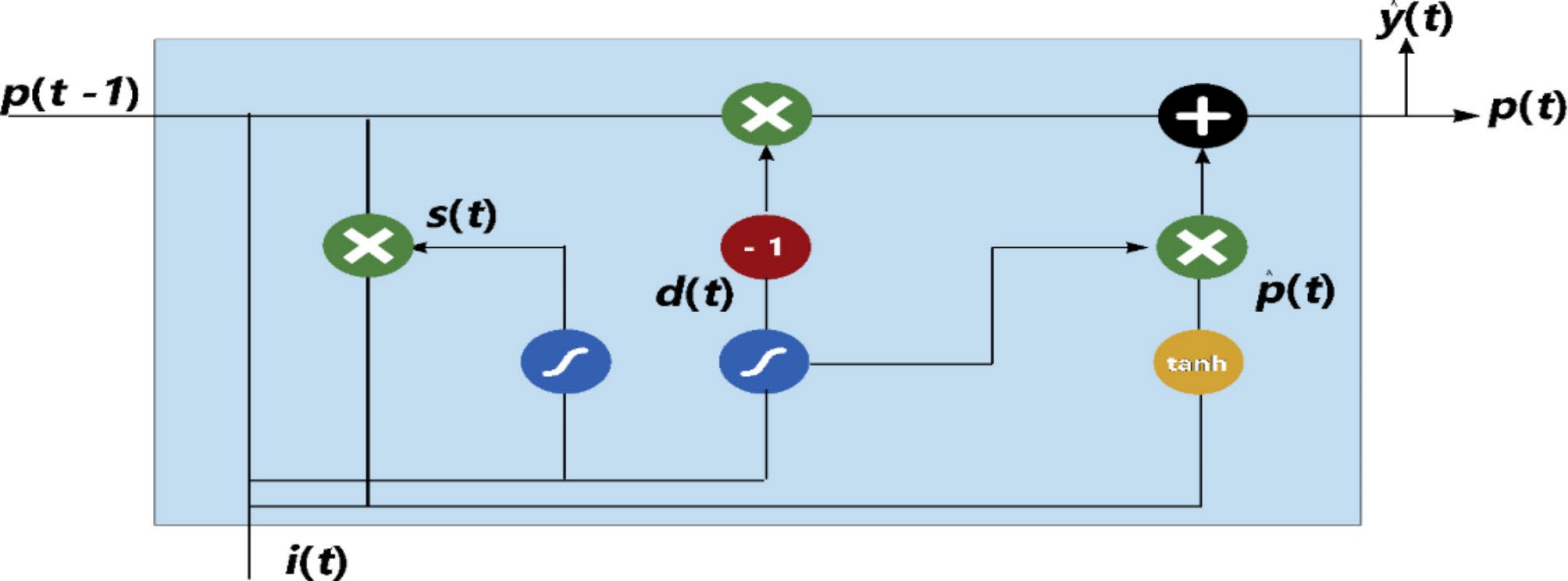
Gated Recurrent Unit structure.

Any input that has already been processed is passed on to the next time step of an RNN, which is referred to as a recurrent neural network. RNN model training is plagued by vanishing and inflating gradient challenges. GRU and LSTM, two cutting-edge deep learning approaches, effectively handle this issue. An entirely new version of RNN, the GRU, has been developed to alleviate training difficulties and to retain the internal state of the system during a repeating process^55^. Cho suggested the gated recurrent unit in 2014 as a variant of the LSTM. GRU networks are seldom employed in regression issues, and their primary use is in classification. Traditional LSTMs take longer to train and have more parameters than LSTMs since the absence of an output gate^56^. Figure 6 depicts the network topology of gated recurrent units.

The relationship between output and input can be summarized as in Eqs. (6)–(9).

6 $$s\left( t \right) = \sigma _{g} \left( {M_{s} i\left( t \right) + U_{s} p\left( {t - 1} \right) + b_{s} } \right)$$

7 $$\:d\left(t\right)={\sigma\:}_{g}({M}_{d}i\left(t\right)+{U}_{d}p\left(t-1\right)+\:{b}_{d}\:)$$

8 $$\:p\left(t\right)=\left(1-d\left(t\right)\right)ʘ\:p\left(t-1\right)+d\left(t\right)\:ʘ\:\widehat{p}\left(t\:\right)$$

9 $$\:\widehat{p}\left(t\:\right)=\:{\sigma\:}_{h}({M}_{p}i\left(t\right)+{U}_{p}(s\left(t\right)\:ʘ\:p\left(t-1\right))+\:{b}_{p}\:)$$

where *i(t)*, *p(t − 1)*,* s(t)*,* d(t)* are vectors of input, previous output, reset gate, and update gate, respectively. M and U are variable matrices and vectors. There are two types of functions: sigmoid functions *σ*_*g*_, and hyperbolic ones *σ*_*h*_.

The rationale for applying a Gated Recurrent Unit (GRU) model to the PIMA Indian Diabetes Dataset (PIDD), despite its lack of time-dependent structure, lies in the model’s ability to effectively capture complex relationships among the features: Pregnancies, Glucose, BloodPressure, SkinThickness, Insulin, BMI, Age, and DiabetesPedigreeFunction. While GRUs are typically used in sequential data contexts, their gated architecture can also excel in handling feature interdependencies that are not explicitly temporal. In this case, the GRU can identify and retain important interactions among the PIDD features, selectively filtering information to optimize classification performance. By using a GRU, the model is able to capture intricate patterns within the dataset, contributing to enhanced predictive accuracy for diabetes classification, even without a temporal sequence^57–59^. To address the challenges of vanishing and exploding gradients during GRU training, specific techniques were applied. Gradient clipping was used to limit excessively large gradients, preventing instability and ensuring smoother learning. Layer normalization was implemented within the GRU layer to maintain a consistent activation distribution, which helps manage vanishing gradients and stabilize training. Additionally, proper weight initialization was applied to further support gradient propagation across layers. These modifications collectively improved the GRU model’s training stability and allowed it to effectively capture complex feature interactions in the PIMA Indian Diabetes Dataset for accurate diabetes classification.

### Hybrid RFE-GRU model

**Fig. 7:**
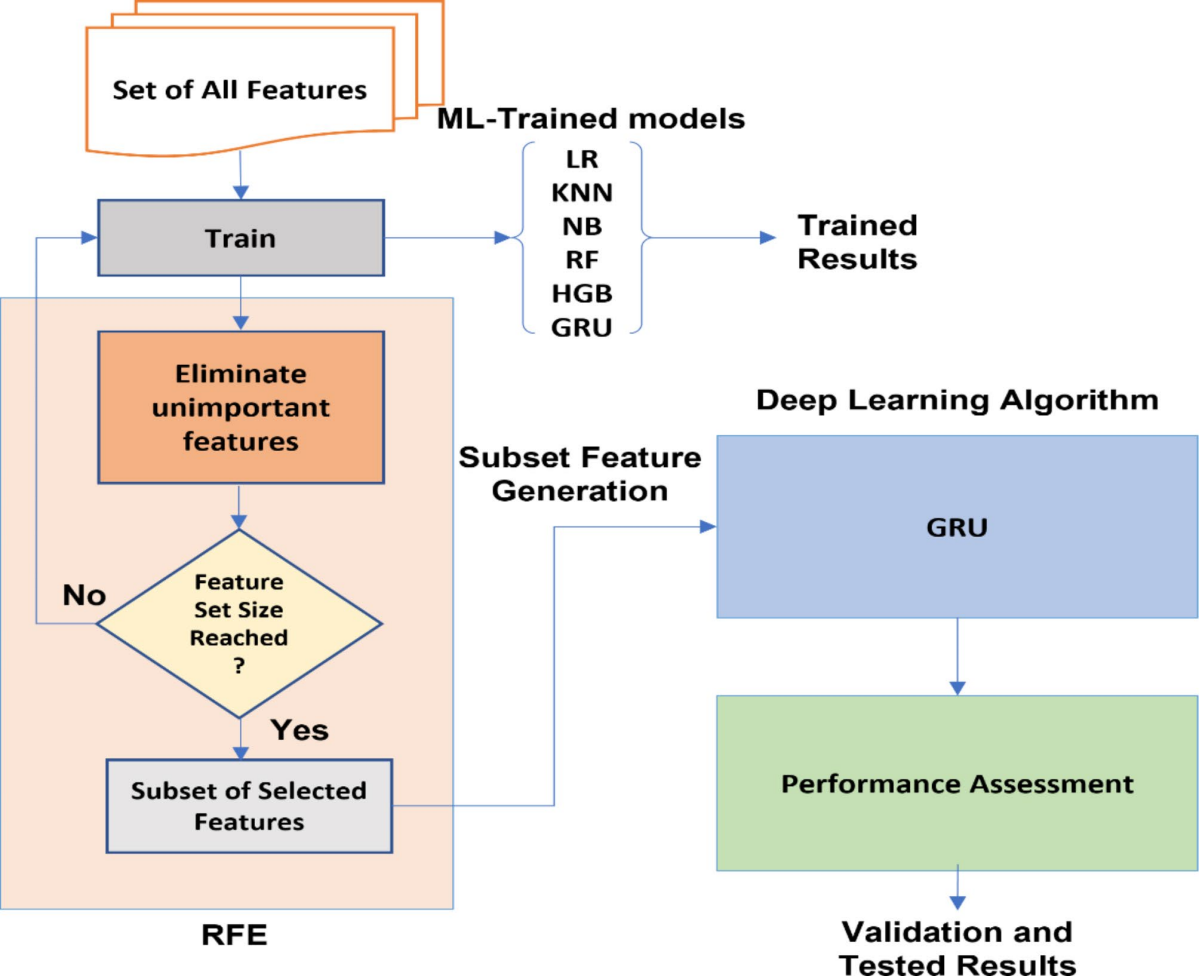
The general block diagram of the proposed RFE-GRU hybrid model.

The main idea of the proposed sequential hybrid RFE-GRU model is to obtain the most relevant features extracted from the PIDD dataset. By training the set of all features to produce feature vectors, these features are acquired using RFE. The vectors are then enrolled until they reach the stage when the extraneous characteristics are removed. Therefore, a choice to select the feature set size based on the RFE is made and then repeated until the accuracy value is fulfilled. Then, as illustrated in Fig. 7, a subset of chosen features (si) is created and enrolled in the GRU model. The approach used by the GRU is comparable to that of an LSTM or RNN, but differs in that it takes as inputs i(t) and the hidden state Pt-1 from the timestamp t-1 before it at each timestamp t. The next timestamp receives a new hidden state Pt, which is returned by the function. Currently, a GRU cell mostly has two gates as compared to an LSTM cell’s three gates^60^. The Reset gate is the first gate, while the Update gate is the second. The network’s hidden state (Pt), or short-term memory, is controlled by the reset gate. The hidden state Pt in GRU may be found in two stages. Producing the ‘candidate hidden state’ is the initial stage. It takes as input both the revealed state from the previous timestamp t-1 and the input, which is multiplied by the output of the reset gate, rt. The ta received this whole information after that. If rt is equal to 1, it means that all of the data from the previous hidden state Pt-1 is being looked at. The information from the previous hidden state is completely disregarded if rt has a value of 0. The current hidden state Pt is then created using the candidate state. The Update gate is useful in this situation as shown in Algorithm 1 which involving recursive feature elimination and GRU.

**Algorithm 1:**
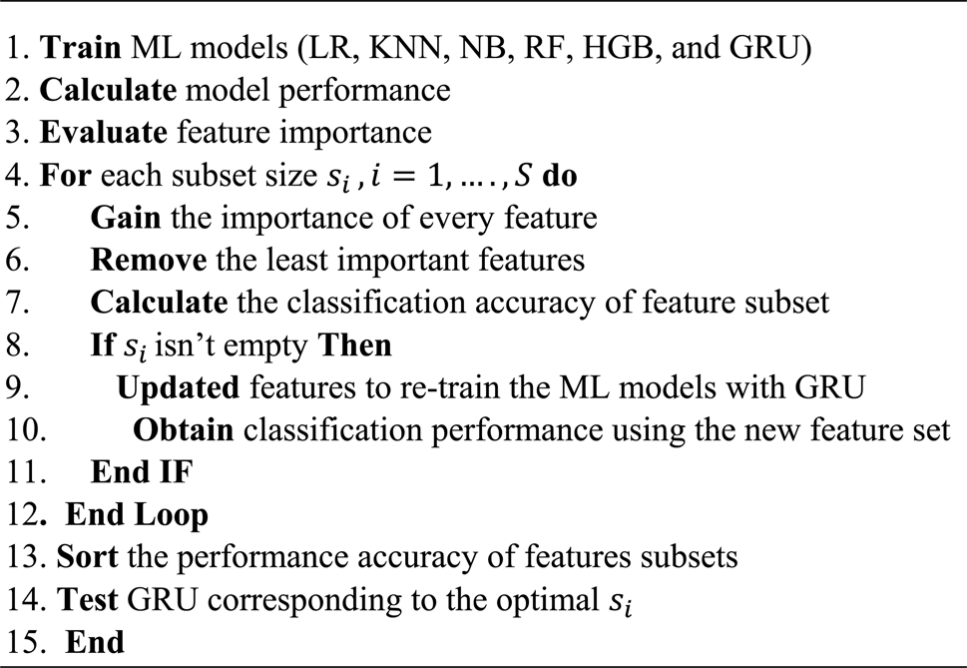
Proposed RFE- GRU hybrid model

RFE starts by training the chosen machine learning algorithm (GRU), sometimes referred to as the core model, with all of the features included in the training dataset. The characteristics are then ordered according to their scores in decreasing order. The characteristics are ranked from most essential to least important in this procedure, with those with higher significance scores being valued more highly for the performance of the model. This feature ranking by RFE reveals the relative importance of each feature, assisting in the selection of the GRU algorithm’s most pertinent characteristics. RFE then purges the dataset of the feature(s) with the lowest significance score. In this stage, the least significant features are successively eliminated to provide a more refined feature subset that only contains the most useful properties for the GRU algorithm. RFE is an iterative procedure that doesn’t stop until either a stopping condition is met, or a certain feature set size limit is reached. The top-ranked features that have survived the iterative elimination process are then combined to form the final feature set. The least valuable features are regularly removed by RFE across the iterations, thereby shrinking the size of the feature set. By concentrating on the most pertinent characteristics for the particular job at hand, the final feature set produced by RFE contributes to improving the model’s interpretability, lowering computational cost, and enhancing its generalization capabilities.

## Experiments and discussions

### Datasets

**Fig. 8:**
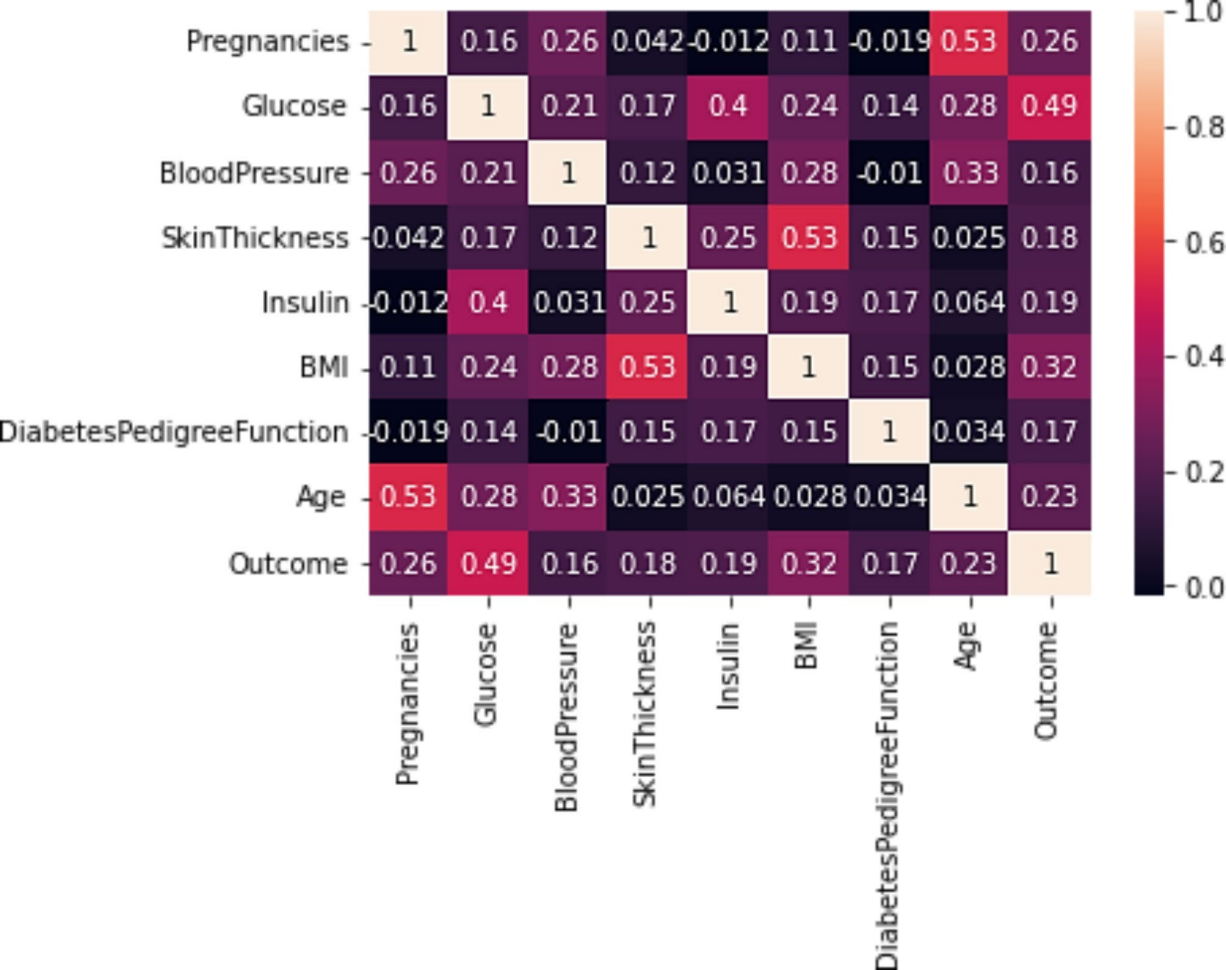
Heatmap analysis for the dataset features.

In this paper, the suggested dataset is collected from the National Institute of Diabetes, Kidney, and Digestive Diseases^61^. The data is available at Kaggle web site as standard dataset: https://www.kaggle.com/datasets/uciml/pima-indians-diabetes-database. PIMA Indians Dataset (PIDD) for testing Gated Recurrent Unit (GRU) is chosen for several compelling reasons. Firstly, PIMA, as a publicly available dataset, offers researchers the convenience of reproducibility and allows them to share their findings more easily. Secondly, PIDD has established itself as a benchmark in studies related to diabetes prediction and healthcare analytics. Researchers often select it to enable effective comparisons with prior works and to set a performance baseline for their proposed architectures. Thirdly, PIDD is widely used in machine learning and healthcare research due to its health-related features, particularly in diabetes prediction tasks. As GRU is specifically designed for sequential data analysis, utilizing the PIDD is highly relevant for evaluating GRU’s performance on sequential information. PIDD dataset is splitted into 80% training and 20% testing. The aim of this study is to classify diabetes disease based on diagnostic data (if a patient has diabetes or not). The selection of these cases from a wider database was constrained by certain limitations. All patients in this study are Pima Indian women at least 21 years old. The main features in this dataset are pregnancies, Glucose, BloodPressure, SkinThickness, Insulin, BMI, DiabetesPedigreeFunction, Age, Outcome. Table 2 illustrates some statistical analysis for the PIDD. Figure 8 shows the heatmap analysis for the dataset features.

**Table 2:** Statistical analysis for the PIDD.

	Count	Mean	Std	Min	Max	25%	50%	75%
Pregnancies	768	4.4	2.95	1.000	17	2	3.84	6.000
Glucose	768	121.4	30.5	44	199	99	117	140.2
BloodPressure	768	72.1	12.1	24	122	64	72	80
SkinThickness	768	26.5	9.7	7	99	20.5	23	32
Insulin	768	118.4	93.7	14	846	79.7	79.7	126.5
BMI	768	32.3	6.9	18.2	67.10	27.3	32	36.5
DiabetesPedigreeFunction	768	0.47	0.33	0.07	2.42	0.24	0.37	0.637
Age	768	33.1	11.78	21	81	24	29	4
Outcome	768	0.34	0.47	0	1	0	0	1

**Fig. 9:**
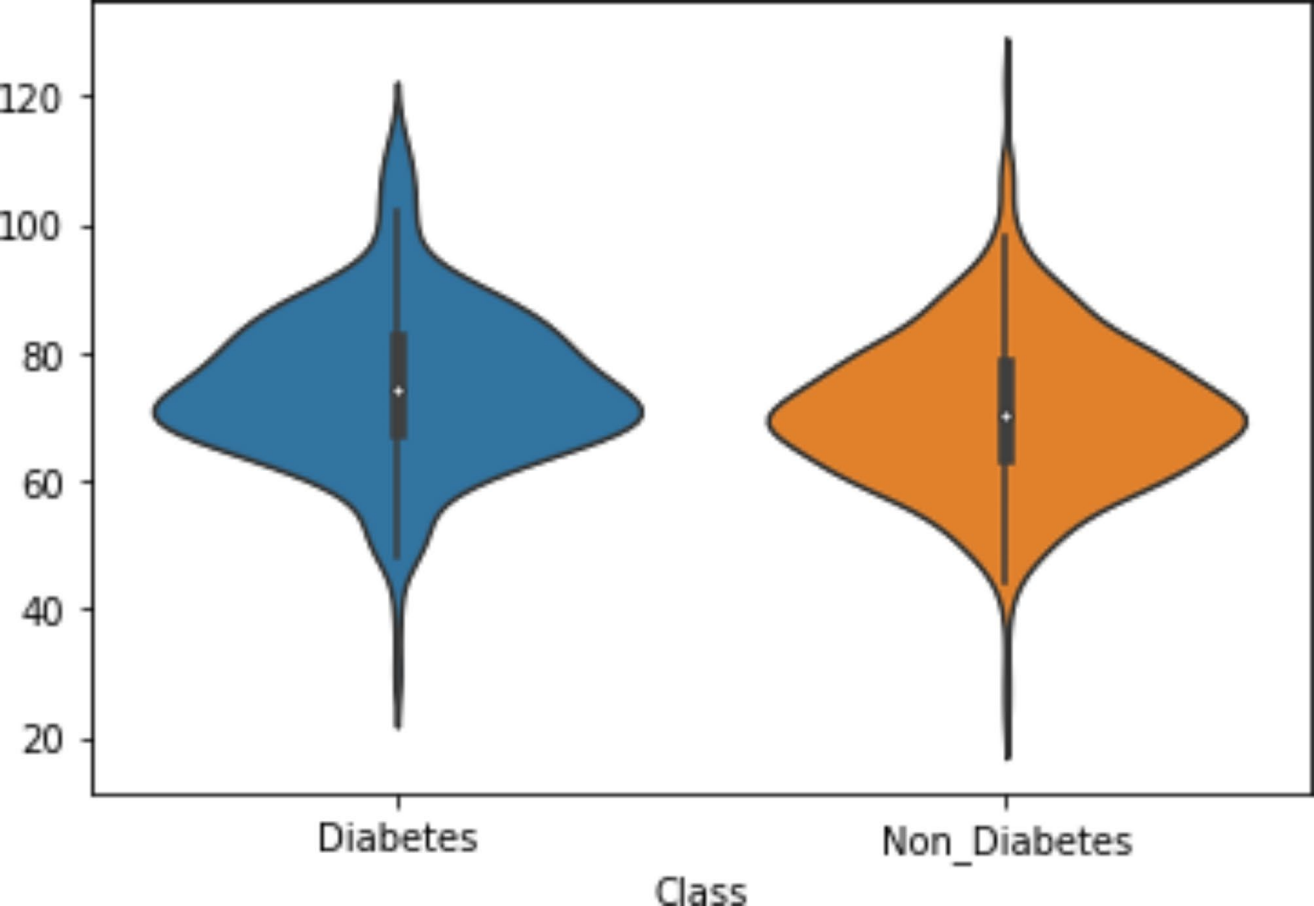
Violin plot visualization per category label.

**Fig. 10:**
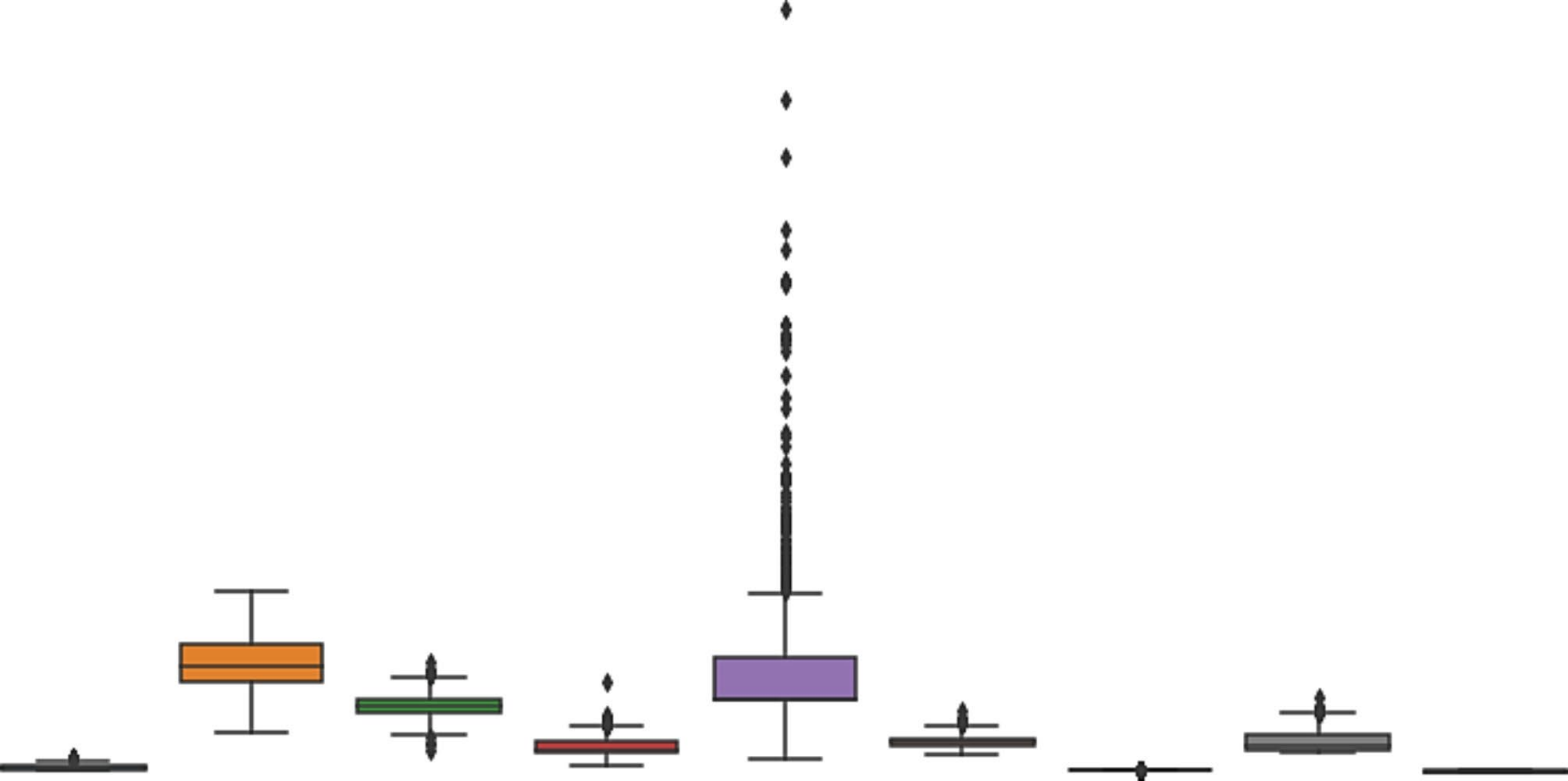
Boxplot visualization for the dataset features.

Figure 9 demonstrates the violin plot visualization per category label. Figure 10 shows the boxplot visualization for the dataset features.

Statistical significance tests were performed to ensure that the observed improvements in the RFE-GRU model were not due to chance. Alongside visual analyses like heatmaps (Fig. 8), violin plots (Fig. 9), and boxplots (Fig. 10) for the PIMA Indian Diabetes Dataset (PIDD) features, we conducted statistical tests to validate the model’s performance improvements. In the future we intended to utilize metrics such as accuracy and AUC were compared against other models using paired statistical tests, like the paired t-test or Wilcoxon signed-rank test, to confirm that the RFE-GRU model’s enhancements in classification performance were statistically significant. This rigorous statistical analysis supports the robustness of the model’s results.

### Evaluation metrics

Evaluation metrics are used in two phases in data categorization problems: the training phase and the testing phase. In the training phase, the evaluation measure was used as a discriminator to identify and choose the best solution for predicting future assessment of a certain classifier with greater accuracy. In the testing phase, evaluation metrics were used as evaluators to determine how successful the classifier was when applied to unknown data^62^.

#### Accuracy

 The accuracy of a dataset is measured by the proportion of predictions that are right as in Eq. (10).

10 $${\text{Accuracy}}\left( {{\text{acc}}} \right) = \frac{{{\text{TP}} + {\text{TN}}}}{{{\text{TP}} + {\text{FP}} + {\text{TN}} + {\text{FN}}}}$$

where $\:TP$, $\:TN$, $\:\text{F}\text{P}$, $\:\text{F}\text{N}$ are true positive, true negative, false positive, false negative values of the confusion matrix, respectively.

#### Precision

 The positive patterns in a positive class that are accurately predicted from all the total anticipated patterns are measured by precision as in Eq. (11).

11 $${\text{Precision}}\left( {\text{P}} \right) = \frac{{{\text{TP}}}}{{{\text{TP}} + {\text{FP}}}}$$

####  F1 score

Precision and sensitivity are the two most important metrics that make up this score as in Eq. (12).

12 $${\text{F}}1{\text{Score}} = \frac{{2{\text{TP}}}}{{2{\text{TP}} + {\text{FP}} + {\text{FN}}}}$$

####  Recall

Recall is the percentage of positive patterns that are properly categorized as in Eq. (12).

13 $${\text{Recall}}\left( {\text{r}} \right) = \frac{{{\text{TP}}}}{{{\text{TP}} + {\text{TN}}}}$$

####  Area under the curve (AUC)

As a prominent ranking statistic, AUC was utilized to build an optimal learning model, as well as to compare learning approaches. AUC value reflects the overall ranking performance of a classifier as in Eq. (14).

14 $${\text{AUC}} = \frac{{S_{p} - N_{p} + (N_{n} + 1)/2}}{{N_{p} N_{n} }}$$

where $$\:{S}_{p}$$ denotes the ratio of the successfully categorized applied negative class instances, while $$\:{N}_{p}$$$$\:{\text{a}\text{n}\text{d}\:N}_{n}$$ represent the number of positive and negative classes, respectively.

### Results and discussion

The experiment results were developed, written, and executed in Python 3.8 using the jupyter notebook version (6.4.6) with Intel Core i5 and 16 GB RAM using Microsoft Windows 10 × 64-bit. Jupyter notebook facilitate the using and writing python codes, where jupyter notebook is an open source utilized for constructing and execution of several machine learning models for both classification and regression. The optimal number of features is the final feature set size. The selected features by RFE are, Glucose, BloodPressure, Insulin, and BMI for GRU model. In GRU model, the number of hidden units is 64. The batch size is 32, the learning rate used is 0.01, number of epochs are 200, the optimizer used is Adam optimizer, the time steps are 8, and the activation function used in the output is sigmoid activation function. To evaluate the performance of RFE-GRU model for diabetes classification, five classifiers’ models, namely, LR, RF, HGB, KNN, and NB are used for the comparison. The performance of these classification models was evaluated using the metrics, namely, accuracy, precision, recall, F1 score, and AUC. Table 3 illustrates the configuration of the hyperparameters for models, namely, LR, RF, HGB, KNN, and NB used in this study. Each model has specific hyperparameters that determine its behavior and performance during training and prediction. Proper tuning of these hyperparameters is crucial to achieve the best results for each model on a given machine learning task and dataset. For LR model, the penalty is 12 that is known the regularization term or ridge regularization, and fit_intercept is true (to calculate the intercept for the model). For RF model, n_estimators are 100 (the number of decision trees in the forest). For HGB model, the learning_rate is 0.01 (the step size at which the algorithm makes corrections). For KNN model, n_neighbors are 5 (the number of nearest neighbors to consider during prediction), and the distance is Euclidean (the distance metric used to calculate the distance between data points). For NB model, alpha is 0.5 (the additive smoothing parameter, also known as Laplace smoothing), and fit_prior is true (whether to learn class prior probabilities from the data).

**Table 3:** Specification of the hyperparameter for the machine learning models.

Models	Parameters
LR	Penalty = l2, fit_intercept = true
RF	N_estimators = 100
HGB	Learning_rate = 0.01
KNN	N_neighbors = 5, distance = Euclidean
NB	Alpha = 0.5, fit_prior = true

Table 4 demonstrates the experimental results of accuracy, F1 score, recall, precision, and AUC for the models, namely, GRU, LR, RF, HGB, KNN, and NB, respectively, without applying RFE.

**Table 4:** Comparison of prediction performances between the classification models without applying RFE.

Models	Accuracy	F1 score	Recall	Precision	AUC
LR	81.76%	81.84%	81.91%	81.64%	0.8841
RF	83.81%	83.16%	83.52%	83.28%	0.8894
HGB	78.64%	78.91%	78.65%	78.51%	0.8435
KNN	73.72%	73.49%	73.20%	73.81%	0.5165
NB	68.54%	68.52%	68.69%	68.39%	0.5074
GRU	87.65%	87.61%	87.62%	87.98%	0.8974

Table 4 presents the performance of several experimental models, with the GRU model achieving the highest accuracy, F1 score, recall, precision, and AUC, at 87.65%, 87.61%, 87.62%, 87.98%, and 0.8974, respectively. On the other hand, the NB model yields the poorest results, with an F1 score, recall, precision, accuracy, and AUC of 68.52%, 68.69%, 68.39%, 68.54%, and 0.5074, respectively. The LR model achieves an F1 score, recall, precision, accuracy, and AUC of 81.84%, 81.91%, 81.64%, 81.76%, and 0.8841, respectively. The RF model performs well with an F1 score, recall, precision, accuracy, and AUC of 83.16%, 83.52%, 83.28%, 83.81%, and 0.8894, respectively. Meanwhile, the HGB model achieves an F1 score, recall, precision, accuracy, and AUC of 78.91%, 78.65%, 78.51%, 78.64%, and 0.8435, respectively. The KNN model achieves an F1 score, recall, precision, accuracy, and AUC of 73.49%, 73.20%, 73.81%, 73.72%, and 0.5165, respectively. Table 4 indicates that the GRU model performs better than the LR, RF, HGB, KNN, and NB models without RFE. Additionally, the RFE-GRU model yields the best results among all the models presented in Table 5. The experimental results of accuracy, F1 score, recall, precision, and AUC for the models, namely, RFE-GRU, LR, RF, HGB, KNN, and NB, respectively, are demonstrated in Table 5.

**Table 5:** Comparison of prediction performances between RFE-GRU model and other models.

Models	Accuracy	F1 score	Recall	Precision	AUC
LR	85.70%	85.50%	85.70%	85.50%	0.9192
RF	86.40%	85.80%	86.40%	86.10%	0.9187
HGB	80.70%	81.00%	80.70%	81.60%	0.8692
KNN	75.00%	76.00%	75.00%	77.90%	0.5226
NB	69.20%	69.90%	69.20%	70.70%	0.5186
Proposed RFE-GRU	90.70%	90.50%	90.70%	90.50%	0.9278

**Fig. 11:**
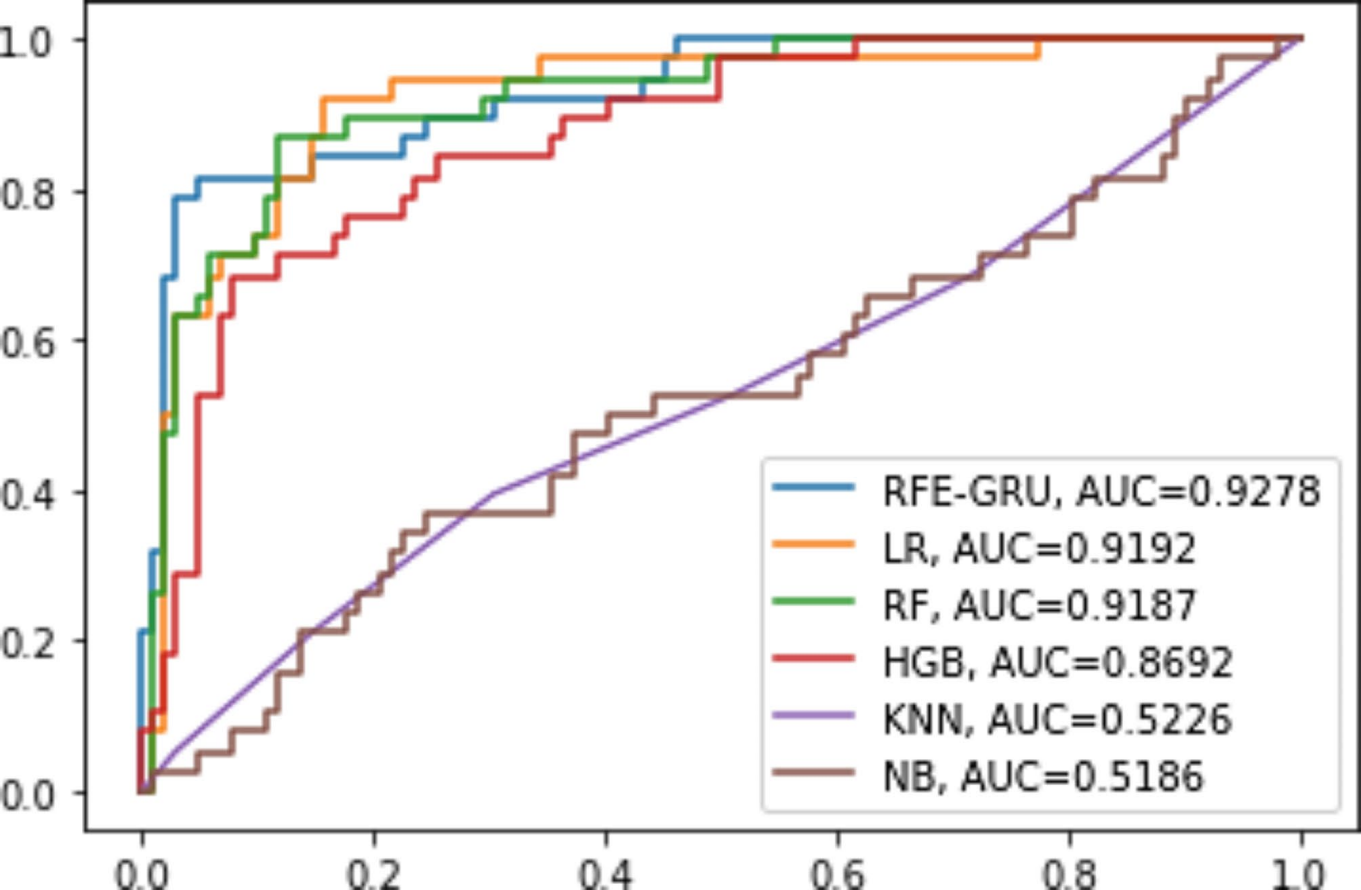
AUC for the models namely, RFE-GRU, LR, KNN, RF, HGB, and NB.

**Fig. 12:**
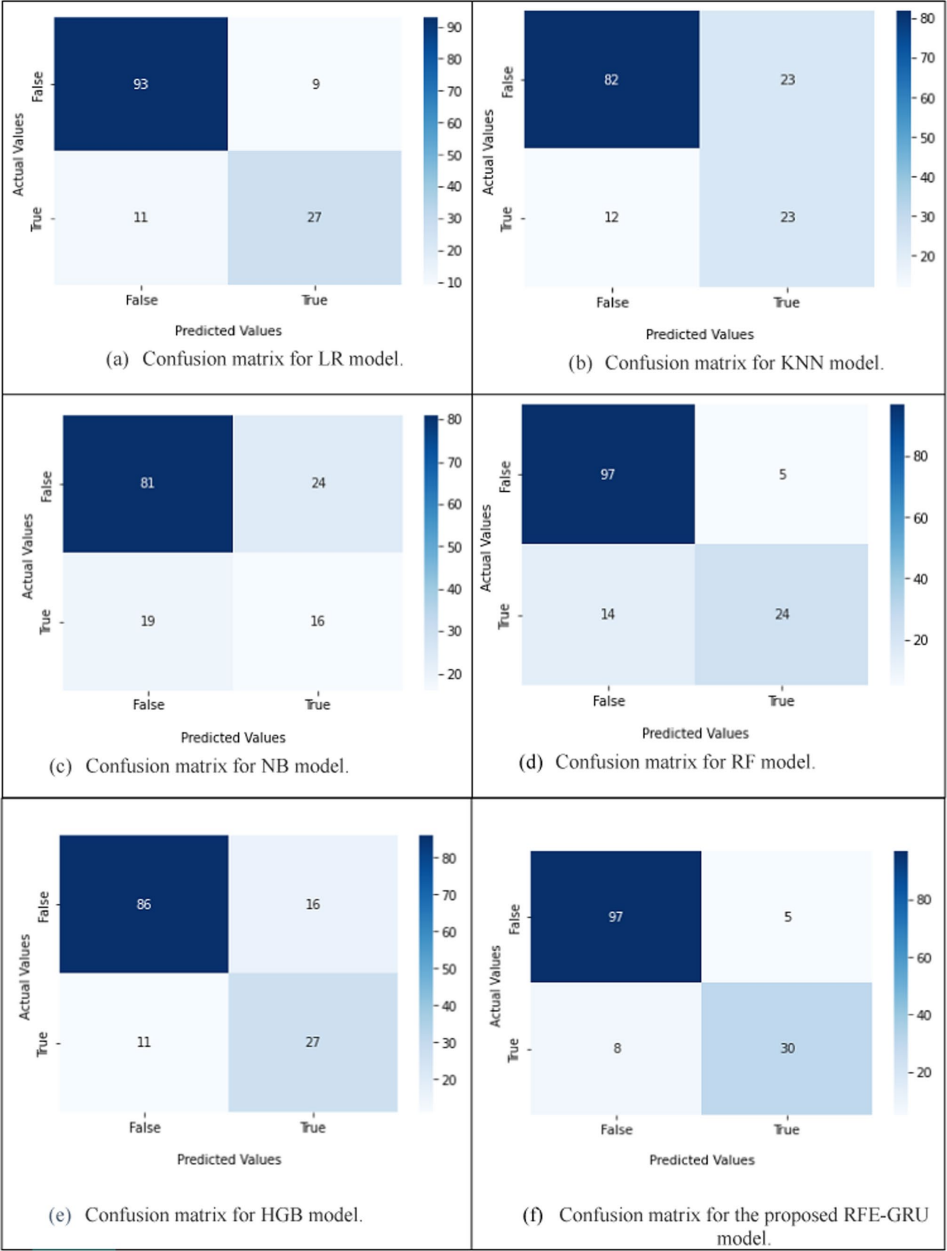
Confusion matrices of (**a**) LR, (**b**) KNN, (**c**) NB, (**d**) RF, (**e**) HGB, and (**f**) the proposed RFE-GRU models.

Among all the experimental models in Table 5, RFE-GRU model gives the best results, its accuracy, F1 score, recall, precision, and AUC are 90.7%, 90.5%, 90.7%, 90.5%, and 0.9278, respectively. The NB model gives the worst results, its F1 score, recall, precision, accuracy, and AUC are 69.9%, 69.2%, 70.7%, 69.2%, and 0.5186, respectively. For LR model, the F1 score, recall, precision, and accuracy, AUC are 85.5%, 85.7%, 85.5%, 85.7%, and 0.9192, respectively. The F1 score, recall, precision, accuracy, and AUC for RF model are 85.8%, 86.4%, 86.1%, 86.4%, and 0.9187, respectively. For HGB model, the F1 score, recall, precision, accuracy, and AUC are 81%, 80.7%, 81.6%, 80.7%, and 0.8692, respectively. The F1 score, recall, precision, accuracy, and AUC for KNN model are 76%, 75%, 77.9%, 75%, and 0.5226, respectively. It can be seen from the data in Table 4 that RFE-GRU model is more effective than LR, RF, HGB, KNN, and NB models. Figure 11 depicts the AUC values for the RFE-GRU, LR, KNN, RF, HGB, and NB models, with the RFE-GRU model achieving an AUC of 0.9278, which is close to 1 and indicates good performance. Figure 12 shows the confusion matrix for the RFE-GRU, LR, KNN, NB, RF, and HGB models.

In this study, the feature set selected by RFE—which includes Glucose, BloodPressure, Insulin, and BMI—was used in the GRU model. This subset of features was found to yield the best results compared to other classification models. To further investigate the impact of RFE on model performance, we conducted an ablation study. This study involved training the GRU model both with and without the selected features identified by RFE, allowing us to directly assess the contribution of feature selection to the overall performance.

Table 4 presents a comparison of prediction performances between various classification models, including GRU, without applying RFE. These models were evaluated using key metrics such as accuracy, F1 score, recall, precision, and AUC, which provide insight into their general performance without the influence of RFE. In contrast, Table 5 compares the performance of the RFE-GRU model with other models after applying RFE. The results clearly demonstrate a significant enhancement in the evaluation metrics, including accuracy, F1 score, recall, precision, and AUC, after incorporating RFE into the GRU model. This improvement underscores the effectiveness of feature selection in boosting the model’s predictive power, showing that the GRU model benefits substantially from a more focused set of relevant features. By isolating the impact of RFE, the ablation study highlights the critical role feature selection plays in refining the model’s performance and robustness, providing a clearer understanding of how each component—both the GRU architecture and the selected features—contributes to the overall classification success.

### Discussion

The likelihood of model biassing is increased in PIDD by the imbalance between normal and abnormal instances. Additionally, PIDD’s total instance count of 768 is insufficient to use deep learning to create a stable model for the hold-out validation approach. Although this strategy is not employed in our experiment, this can be used in cross-fold validation. Despite the significance of PIDD data pretreatment being made clear and documented in the proposed methodology, its significance for developing classification models both before and after the preprocessing was implied. However, the researchers who worked on data processing and developing a deep learning model did not deal with data pretreatment and were only concerned with accuracy, which had a detrimental impact on their confidence in the model’s results when they were used. In this paper, an RNN passes on any input that has already been processed to the following time step. Challenges with disappearing and inflated gradients are common during RNN model training^63–65^. Modern deep learning techniques GRU and LSTM successfully address this problem. To overcome training challenges and maintain the internal state of the system over a repeating process, an altogether new RNN variant called the GRU has been designed. Furthermore, the gated recurrent unit as an alternative to the LSTM are mostly used for classification and are rarely used in regression problems. The results stated that the proposed method gives better results than the state of the art using the same PIDD dataset. To verify the validity of the proposed RFE-GRU model in this paper, the proposed model is compared and analyzed with another several models using PIDD as shown in Table 6.

**Table 6:** Comparative study of several related studies for diabetes classification using PIDD.

Studies	Model	Accuracy
Çalişir and Doğantekin^16^	LDA – MWSVM	89.74%
Dadgar and Kaardaan^17^]	Neural network and genetic algorithm	87.46%
Chen et al.^18^	DT and K-means	90.00%
Haritha et al.^19^	Firefly and cuckoo models	81.00%
Zhang et al.^20^	Feedforward neural network	82.00%
Benbelkacem and Atmani^21^	RF	77.00%
Khanwalkar and Soni^22^	Sequential minimal optimization (SMO)	77.34%
Maniruzzaman et al.^9^	LR	77.06%
Patra and Kuntia^23^	SDKNN	83.76%
Bhoi et al.^24^	LR	76.80%
	Neural networks	75.80%
	RF	75.40%
Ramesh et al.^25^	LR	73.30%
	KNN	79.80%
	NB	73.10%
	SVM + Radial basis function (RBF)	83.20%
Salem et al.^26^	DT	81.89%
	NB	81.89%
	Fuzzy-KNN	90.55%
	TFKNN	90.63%
Proposed RFE-GRU	Recursive feature elimination and gate recurrent unit	**90.70%**

As shown in Table 6, it is seen that the classification accuracy of the proposed RFE-GRU model is the highest. It is shown that the proposed model used in this study solves the shortage of another models and improves the classification accuracy of PIDD.

In this study, we split the PIDD dataset into 80% for training and 20% for testing, and while we did not apply k-fold cross-validation, it is an approach that could be valuable for further validation, especially given the dataset’s small size of 768 instances and the potential bias introduced by the imbalance between normal and abnormal instances. The limited size and imbalance increase the likelihood of model overfitting or underfitting, making cross-validation a useful technique to better assess the model’s generalization ability. Additionally, we recognize the importance of proper data preprocessing, which was not fully addressed in previous studies that focused primarily on model accuracy. Proper handling of the class imbalance and the application of techniques like resampling or synthetic data generation could improve the model’s robustness. Despite these challenges, the GRU model mitigates issues like vanishing and exploding gradients, was successfully applied for diabetes classification, achieving better results than state-of-the-art methods using the same PIDD dataset. For further validation, external datasets and a more comprehensive evaluation involving metrics such as ROC and AUC would be useful to assess the model’s performance in different contexts and populations.

The novelty of the RFE-GRU model lies in its integration of Recursive Feature Elimination (RFE) with Gated Recurrent Unit (GRU) architecture, a combination designed to enhance both the accuracy and interpretability of diabetes classification. Unlike conventional models that either prioritize computational efficiency at the expense of predictive depth or rely solely on feature-rich deep learning architectures, the RFE-GRU model optimizes feature selection to streamline the predictive process, focusing only on the most impactful features—Glucose, BloodPressure, Insulin, and BMI—identified through RFE. This targeted approach not only reduces computational load but also provides insights into the biological markers that most significantly influence diabetes outcomes, fostering greater interpretability.

Furthermore, the GRU component uniquely addresses the limitations of simpler machine learning models, such as Logistic Regression (LR) or Random Forest (RF), which lack the capacity to model sequential data dependencies. GRU’s recurrent structure captures temporal patterns that may reflect underlying trends in physiological data, a dimension crucial for chronic disease modeling but typically overlooked by static classifiers. Additionally, GRU mitigates common deep learning challenges such as vanishing gradients, thanks to its gated architecture, offering a more stable training process than conventional RNNs while maintaining efficient memory usage.

In comparison to previous approaches focused primarily on static feature usage or complex yet computation-heavy architectures, the RFE-GRU model provides a balanced, interpretable, and computationally feasible solution for diabetes prediction. This combination of selective feature importance and sequence-based learning presents a refined classification tool, making it uniquely equipped to outperform traditional models on the PIDD dataset.

### Limitation and future directions

The proposed RFE-GRU model demonstrates strong performance for diabetes classification using the PIDD dataset, but several limitations should be considered when interpreting its results, particularly regarding its application to datasets with different characteristics. One notable limitation is the inherent class imbalance between normal and abnormal instances in the PIDD dataset, which can increase the likelihood of model bias. This imbalance could lead to overfitting or underfitting, especially with deep learning models like GRU, which are sensitive to such issues. Although the study does not employ cross-validation, it is an important strategy to consider in future work to mitigate the impact of this imbalance and assess the model’s generalization ability more robustly.

Another limitation lies in the size of the PIDD dataset, which contains only 768 instances. This relatively small dataset may not provide enough diverse examples for deep learning models to learn stable, generalizable patterns. The use of the hold-out validation approach, which we employed in this study, may not be the most reliable for ensuring a robust model due to this small sample size. Future work could involve applying k-fold cross-validation, which would offer a more reliable estimate of the model’s performance and further reduce the potential for overfitting. Additionally, the dataset’s small size and class imbalance could benefit from techniques like resampling, synthetic data generation, or class weighting to improve model robustness and reduce bias. Furthermore, while the significance of data preprocessing was addressed, previous works often overlooked its importance, focusing mainly on model accuracy. In this study, data pretreatment, particularly handling missing values and scaling, was necessary for improving model accuracy, but more attention should be paid to preprocessing steps to ensure consistent results across various datasets. In terms of model design, although the GRU model overcomes common challenges of RNNs, such as vanishing and exploding gradients, by maintaining the internal state over time, its performance may still vary significantly depending on the characteristics of different datasets. External validation on other datasets with diverse features or different populations would help evaluate the GRU model’s ability to generalize across different contexts, providing a clearer understanding of its strengths and limitations. Finally, future studies could expand the evaluation by incorporating additional metrics like ROC and AUC to assess model performance more comprehensively and validate the model’s robustness in real-world applications.

## Conclusion

The machines learning (ML) models have been praised for their usefulness in making diagnosis for many patients. Several ML classification models currently are used for the prediction and classification of diabetes patients. In this paper, a novel model called Recursive Feature Elimination-Gated Recurrent Unit (RFE-GRU) is constructed in this study for PIDD classification. Different performance metrics, namely, accuracy, F1 score, recall, precision, and AUC were used to evaluate the impact of the RFE-GRU model. The F1 score, recall, precision, accuracy, and AUC for RFE-GRU model are 90.50%, 90.70%, 90.50%, 90.70%, and 0.9278, respectively. The proposed RFE-GRU model was compared with other conventional machine learning models, where RFE-GRU model achieved the best results. The worst results were obtained by NB model, its F1 score, recall, precision, accuracy, and AUC are 69.90%, 69.20%, 70.70%, 69.20%, and 0.5186, respectively. In the future, new machine learning and deep learning models will be applied to this dataset to acquire better results. Furthermore, we plan to use the fuzzy and neutrosophic system with RFE-GRU for biomedical analysis and implement uncertainty features for medical applications.

## Data Availability

The diabetes PIMA Indian dataset (PIDD) was used for classification in several studies, it includes 768 instances and 9 features; eight of the features are the predictors, and one feature is the target. https://www.kaggle.com/datasets/uciml/pima-indians-diabetes-database.
